# Focus on the Protein Fraction of Sports Nutrition Supplements

**DOI:** 10.3390/molecules27113487

**Published:** 2022-05-28

**Authors:** Luisa Pellegrino, Johannes A. Hogenboom, Veronica Rosi, Marta Sindaco, Stefano Gerna, Paolo D’Incecco

**Affiliations:** Department of Food, Environmental and Nutritional Sciences (DeFENS), University of Milan, 20133 Milan, Italy; luisa.pellegrino@unimi.it (L.P.); john.hogenboom@unimi.it (J.A.H.); veronica.rosi@unimi.it (V.R.); marta.sindaco@studenti.unimi.it (M.S.); stefano.gerna@unimi.it (S.G.)

**Keywords:** sport supplements, whey proteins, protein aggregates, amino acids, capillary electrophoresis, confocal microscopy

## Abstract

Increasing awareness of balanced diet benefits is boosting the demand for high-protein food and beverages. Sports supplements are often preferred over traditional protein sources to meet the appropriate dietary intake since they are widely available on the market as stable ready-to-eat products. However, the protein components may vary depending on both sources and processing conditions. The protein fraction of five commercial sports supplements was characterized and compared with that of typical industrial ingredients, i.e., whey protein concentrates and isolates and whey powder. The capillary electrophoresis profiles and the amino acid patterns indicated that, in some cases, the protein was extensively glycosylated and the supplemented amino acids did not correspond to those declared on the label by manufacturers. The evaluation by confocal laser scanning microscopy evidenced the presence of large aggregates mainly enforced by covalent crosslinks. The obtained findings suggest that, beside composition figures, provisions regarding sports supplements should also consider quality aspects, and mandatory batch testing of these products would provide more reliable information to sport dieticians.

## 1. Introduction

Sports supplements are among the fastest-growing products on the market over the last few decades, and their consumption is no longer restricted to athletes only, in line with the increased attention of people for a healthy lifestyle [1]. Notably, the literature in this research area is increasing as well, and the derived scientific knowledge is constantly evolving [2,3]. Sports supplements are basically nutrient-dense foods, with milk protein representing the main component [4]. Minor ingredients are vitamins, minerals, essential fatty acids, selected amino acids, fiber, among others. Milk powder, obtained by drying fresh liquid milk, has been a traditional ingredient due to its large availability and long shelf-stability. However, the more recently available high-protein powders from whey, namely whey-protein concentrates (WPC) and isolates (WPI), are today extensively used in sports supplements, as well as in other nutritional and health preparations and in protein-fortified bars or fermented beverages [5,6]. These products, therefore, lack the casein fraction, which is considered a slow-digesting protein [4,7,8].

Production of WPC and WPI powders starts from either sweet or acid whey [8] that is generally submitted to a thermal treatment to ensure microbiological safety. Then, whey proteins are recovered in their near native form by ultrafiltration (UF), sometimes followed by diafiltration, with partial or total removal of lactose, minerals and non-protein nitrogen, followed by evaporation and spray-drying of the retentate [7]. Basically, WPC and WPI differ in the protein content, which is much higher (>90%) in the latter, due to an extensive removal of lactose before spray-drying [9].

The spray-drying process itself does not pose considerable heat stress to whey protein components, the prior functionalization heat-treatments on the liquid product being mainly responsible for modifications in the protein structure, including denaturation, glycosylation (Maillard reaction) oxidation, and protein–protein interactions [10,11,12]. Whey proteins represent the heat labile fraction of milk proteins as a result of their globular structure stabilized by a variable number of disulfide bonds. The highest thermal stability is reported for α-Lactalbumin (α-La), owing to its capability of binding Ca ions and the lack of free SH-groups. Consistently, Guyomarc’h et al. [13] reported that, in milk heated at 75–90 °C, the denatured β-lactoglobulin (β-Lg) molecules rapidly aggregated, both with other whey proteins and with κ-casein, and the resulting aggregates were found to be exclusively due to disulfide bonds. Small amounts of α-La and bovine serum albumin were found in the aggregates only at higher temperatures. These interactions are irreversible and actually initiate the formation of stable heat-induced aggregates in milk [11,14]. Provided that heating conditions and pH of the liquid whey are sufficiently mild, large amounts of native whey proteins can be present in WPC even after spray-drying [15]. Protein modifications induced by heat treatments increase the protein tendency to further crosslink during the subsequent storage period of the powdered products, thus leading to formation of large insoluble aggregates. The Maillard reaction is responsible for protein glycosylation in products containing lactose or other sugars, whereas, in sugar-free products, crosslinks such as the dehydroalanine-derivatives lysinoalanine and lanthionine, isopeptides, or oxidation products, prevail depending on processing conditions [12,14]. Colantuono et al. [16] have recently demonstrated that large aggregates involving casein micelles can persist in long-stored SMP after rehydration and are unaffected by microbial proteases activity. Aggregation phenomena may also involve fat globules, since milk homogenization provokes the adsorption of both denatured whey proteins and casein micelles to the milk fat globule membrane, thus inducing the formation of fat-protein clusters [17].

Nutritional benefits or issues related to sports supplement consumption are extensively studied [2,18,19], while relatively few studies have been published on the quality of the protein ingredients in these products in relation to the chemical modifications induced by the manufacturing process [9,20,21]. However, sports supplements are increasing in popularity near different consumer categories and can represent a primary protein source in the everyday food. From a technological point of view, the presence of denatured and aggregated proteins affects powder properties, such as solubility and storage stability. Therefore, a more specific focus on the characteristics of this fraction would be highly informative.

The goal of the present work was to characterize the protein fraction of five commercial sports supplements. The selected supplements were representative of the aroma-free types available on the market. In order to achieve greater insights on the status of the protein fraction as affected by process operations, three major industrial ingredients of the sports supplements, i.e., WPC, WPI and whey powder, were taken as reference samples. This was considered a useful approach to understand how and whether whey protein characteristics in the ingredients might have changed in the finished products. Various investigation approaches were adopted, including confocal laser scanning microscopy (CLSM), to shed light on the microstructure of these products, with particular emphasis on the presence of protein aggregates and stable interactions between protein molecules and fat globules. This information will be of practical use to manufacturers of sports supplements when formulating their products using high-protein dairy powders. Suitable parameters based on the evaluation of processing-induced modifications to test upon ingredient selection will be suggested. Furthermore, the quality of protein ingredients in sports supplements should be considered when stating provisions regarding the use of these food products.

## 2. Results and Discussion

### 2.1. Samples Analysed

Five powdered sports supplements (PSS) (three different batches each) were purchased from local suppliers. For confidentiality reasons, brand and manufacturer names were not disclosed. Supplements were labelled exactly as follows: whey proteins obtained by ultrafiltration (A47); hydrolyzed whey protein isolate (C64); whey protein concentrate (Y08); whey protein isolate added with alanine and glutamine (S15); isolated and ultrafiltered whey proteins (L03). The labelled gross composition is compiled in Supplementary Table S1. No protein sources other than dairy were declared in the ingredient list. In addition, three types of ingredients for PSS, i.e., whey protein isolate (WPI), 35% whey protein concentrate (WPC), and whey powder (WP), were supplied (four different batches each) by national importers and used as reference samples.

The composition of the analyzed samples is displayed in Table 1. Despite some variability in the data, due to the different origin of the samples, composition of the reference samples met expectations according to the respective manufacturing process and was in accordance with average literature data [19,20]. Differently, data for individual PSS were less variable and consistent with those labelled on the respective commercial packs (Supplementary Table S1), indicating the adoption of a standardized production. The protein content ranged between 66.5 and 95.8 g/100 g and only one out of the five samples showed a verified protein content >90%, which is the minimum value expected for WPI [8,9]. Similar to WPC and WP, samples A47, C64 and Y08 (whey protein concentrate) contain a small amount of lipids (1.2–3.3%) and of carbohydrates (0.5–6.7%), consisting of maltodextrin in the case of sample C64. These natural polysaccharides are common ingredients in protein powders since they are able to form colorless protein-glycoconjugates that do not undergo the advanced Maillard reaction while improving stability of the rehydrated powder [22]. The moisture contents varied from 3.1 to 7.3%, although all packs were sealed on purchase.

### 2.2. Capillary Zone Electrophoresis of the Protein Fraction

**Figure 1:**
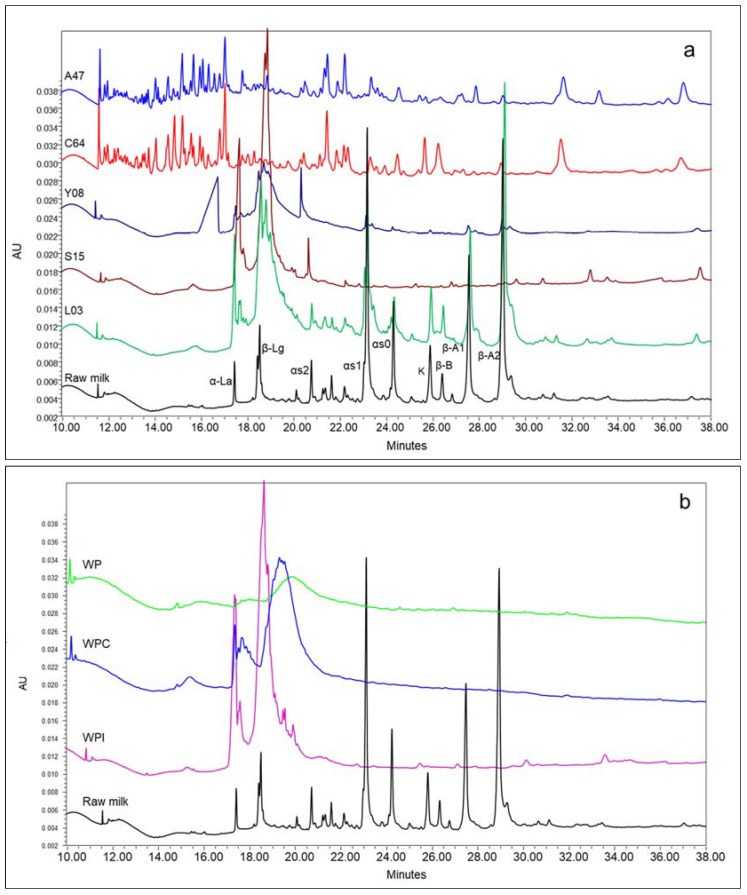
Capillary electrophoresis patterns of: (**a**) commercial sport supplements and; (**b**) control samples. A47: whey proteins obtained by ultrafiltration; C64: hydrolyzed whey protein isolate; Y08: whey protein concentrate; S15: whey protein isolate added with alanine and glutamine; L03: isolated and ultrafiltered whey proteins; WP: whey powder; WPC: whey protein concentrate; WPI: whey protein isolate. Raw milk sample is used as reference for peak identification.

The characteristics of the protein fraction in the commercial samples of PSS were studied and compared with those of three industrial whey-based powders that were taken as reference samples since they represent primary ingredients in PSS. As it was mentioned above, protein molecules in processed whey products may undergo denaturation and cross-linking reactions that have a notable impact on their solubility and thus may impair their determination and quantification. Prior to capillary zone electrophoresis (CZE) analysis, the samples were treated for 4 h with a buffer containing urea as dissociating agent, to suppress non-covalent interactions, and dithiothreitol (DTT) as a reducing agent. Electropherograms of the five PSS are shown in Figure 1a, where the pattern of a raw milk sample is displayed as a reference for correct peak identification. Peaks of native α-La and β-Lg were clearly recognizable for samples L03, S15 and Y08, although both proteins migrated as multiple broad peaks. Such a peak shape indicates that the individual proteins were extensively glycosylated, as previously reported by other authors [23,24,25]. Gasparini et al. [11] observed a direct correlation between the content of residual lactose in the whey protein powders and their glycation. Patterns similar to ours were obtained by Feng et al. [25] in the CZE of skim milk powders and powdered infant formula, and peak broadening was common to both whey proteins and casein peaks. Differently from our conditions, samples were denatured with SDS and reduced with 2-mercaptoethanol. This suggests that the observed electrophoretic behavior of modified proteins is not influenced by the sample preparation conditions. In accordance with this interpretation, peak broadening was greatest in sample Y08, which displayed a high content of lactose (6.7 g/100 g), also indicated in the commercial label. Furthermore, the total peak area of whey proteins in this sample was remarkably lower than in the other samples, probably due to the lower protein content (66.5 g/100 g). The large fronting peak migrating at 15 min has not been identified yet. The electropherograms of both samples A47 and C64 present a series of peaks likely resulting from the hydrolysis process of the whey proteins. Hydrolysed whey proteins are less commonly used in PSS formulation since their physiological advantages are still under debate [26]. Furthermore, hydrolysis makes addition of cheaper proteins undetectable, unless proteomic approaches are implemented [27]. It should be mentioned, however, that the presence of hydrolyzed whey protein was indicated on the label of the PSS C64 only. Unexpectedly, the CZE profile of sample L03 evidenced the presence of casein, in contrast with the commercial label reporting isolated and ultrafiltered whey proteins only. The peaks of all the casein fractions displayed a shape quite similar to those in the reference raw-milk sample, indicating they underwent a limited or no glycosylation. The level of glycosylation was much greater in the whey proteins, suggesting that casein and whey proteins in this PSS derive from two different sources. The absence of lactose and the high content of ash of L03 would suggest the addition of sodium or calcium caseinate, both having the advantage of a high solubility. The electropherograms of reference samples (Figure 1b) confirm that native α-La and β-Lg were still present in the WPI sample, where no lactose was detected, whereas both WP and WPC displayed almost only glycosylated proteins eluting as broad peaks. Much effort has been made to separate milk proteins individually in powdered products, in order to understand the modifications these underwent and to achieve a reliable quantification. Actually, SDS-PAGE is widely used as it does not require expensive equipment. However, large protein aggregates fail to migrate into the gel and the analysis only provides a semi-quantitative estimation [28,29]. Recent applications reported by literature support the reliability of CZE under reducing conditions in the separation of both native and denatured whey proteins [25,30,31], although the incomplete solubilization of large aggregates may also represent a limitation with this technique, as discussed below. Liquid chromatography mass spectrometry methods based on a multiple reaction monitoring approach were recently proposed for the quantification of several milk proteins simultaneously [32,33]. These methods might improve the analysis of aggregated proteins since they include a preliminary digestion of the sample with tripsin.

### 2.3. Amino Acid Composition

The profile of total amino acids (TAA) of all the samples submitted to acid hydrolysis is reported in Table 2. Due to the hydrolysis conditions, Cys and Met were determined as Cysteic acid and Methionine sulphone, respectively, while Trp was not determined. Furthermore, the content of individual free amino acids (FAA) was determined after powder dissolution and deproteination. The data are shown in Table 2 only when FAA are present. The differences were evaluated by one-way ANOVA and results are shown in Supplementary Table S2. The amount of TAA, expressed as g/100 g product, did not always correspond to the protein content reported in Table 1. An incomplete recovery was expected considering the partial degradation of some amino acids (e.g., Trp, Tyr) during acid hydrolysis and the partial loss of Lys due to the irreversible glycosylation during product manufacturing and storage [4]. Gasparini et al. [11] also reported a recovery of TAA after acid hydrolysis of WPC that was 25% lower than the protein content determined by the Kjeldahl method. Surprisingly, the amount of TAA in sample Y08 only accounted for 65% of the protein content. This would suggest the undeclared presence of nitrogen-rich compounds (e.g., urea) to increase the Kjeldahl-assessed protein content. The profile of FAA of this PSS showed the presence of added Gly (2.6%), also not declared in the label. On the other hand, sample S15 contained over 21% of FAA. Although the addition of Gln and Ala was mentioned in the ingredient list, comparable undeclared amounts of free Gly and Lys were also detected in this PSS. Gln supplementation in PSS is rather common due to the known effect against development of exercise-induced fatigue through several mechanisms [9,34]. Traces of free Leu and Tyr detected in sample C64 probably resulted from the enzymatic hydrolysis process the protein ingredient of this PSS underwent. The presence of casein observed in sample L03 by CZE (Figure 1a) accounts for the higher content of Glu, Ser, Pro, Arg, and the lower content of Ile and Asp of this PSS compared to the reference WPI sample, having a similar content of TAA, and depending on the differences in the amino acid composition existing between casein and whey proteins [35]. The latter are routinely present in PSS due to their high-quality amino acid profile. The functional role of individual amino acids in sport nutrition is well documented [9,36]. Additionally, it must be considered that bioavailability of amino acids is largely influenced by both the method of processing and the storage conditions of these products on a shelf. Although these aspects are not addressed in this study, it has been evidenced that the characteristics of PSS could be different from those reported on the label.

### 2.4. Confocal Laser Scanning Microscopy

**Figure 2:**
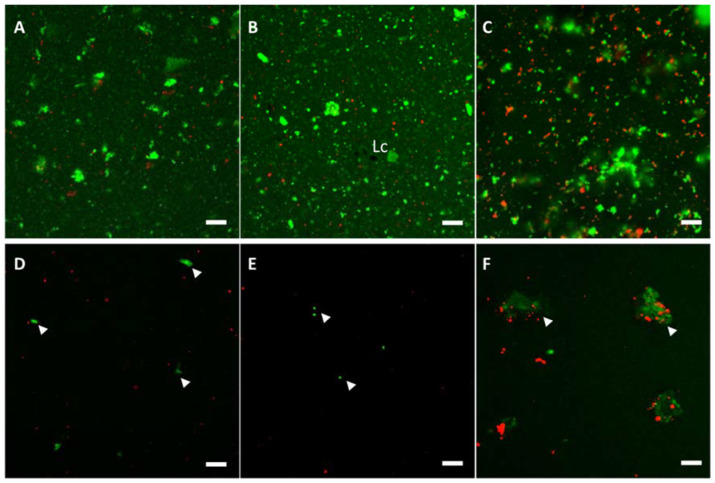
CLSM of whey powder (WP) (**A**), whey protein concentrate (WPC) (**B**) and Y08 (**C**) samples rehydrated with water (upper row) and subsequently solubilized with urea/DDT buffer (lower row). The effect of the buffer is evident comparing (**A**–**C**) panels with the corresponding (**D**–**F**) panels. Arrowheads indicate insoluble aggregates. Protein is green and fat is red. Lc: lactose crystal. Bars are 10 µm.

As mentioned above, concentrated urea solution interrupts hydrogen bonding while DTT reduces covalent disulfide bridges occurring at the inter- and intra-molecular level in protein molecules. Therefore, the protein fraction of the samples was expected to dissolve under the adopted conditions since it comprises whey proteins only. In order to verify the effectiveness of these conditions, we adopted CLSM using a specific sample staining procedure to visualize the protein in green and fat in red. Observations were carried out on WP, WPC and Y08 samples that were only rehydrated with water and, in addition, on the same samples further treated with the urea/DTT buffer (Figure 2). The effect of protein solubilization of the buffer was clearly evidenced in all samples by a remarkable reduction of the green background fluorescence. However, some fluorescence was still detected, and this was ascribable to protein aggregates including fat globules. Notably, the aggregates were few microns in size in both WP and WPC samples (Figure 2D,E) while they were much bigger in Y08 sample (Figure 2F). These structural differences can be explained by the different compositional traits of the samples. In fact, the lactose-to-protein ratio was 5.8 and 1.4 in WP and WPC, respectively, and 0.1 in Y08 (Table 1). In the presence of a high lactose-to-protein ratio, the Maillard reaction prevails over β-elimination reactions, as evidenced by the high levels of furosine and low levels of lysinoalanine observed in milk powders [10]. Indeed, the first products of the Maillard reaction are protein-lactose conjugates that make lysine residues unavailable to form crosslinks [37]. Thus, we could speculate that the lower aggregation level observed in WP and WPC was due to the prevalence of protein glycosylation (Maillard reaction) that did not bring extensive covalent crosslinking, at least at the heating conditions attained in manufacturing, and thus these products were almost completely solubilized by the urea/DTT buffer that dissociated the disulfide bridges. On the contrary, the low lactose content in Y08 kept the Maillard reaction extent low, so allowing the formation of irreversible crosslinks between proteins resulting in large aggregates. Both the size and shape of whey protein aggregates can vary from microgel to strands or to globular aggregates depending on pH, ionic strength, nature of salts [38,39]. Furthermore, the formation of aggregates was favoured by the presence of fat, which was higher in Y08. Moreover, Wang and Lucey [40] observed the presence of larger protein aggregates in WPC than in WPI, as an effect of the higher fat content (up to 10%) in the former.

**Figure 3:**
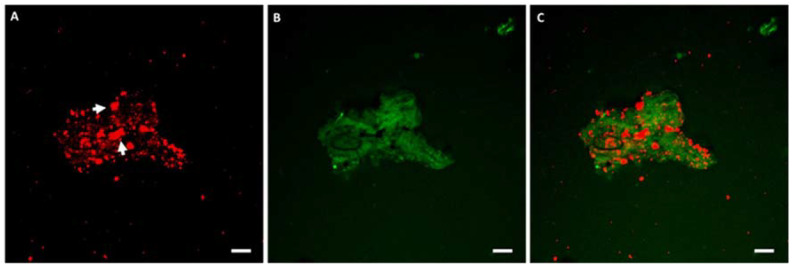
CLSM of a protein aggregate in urea/DDT buffer. Separate fluorescent channels for fat (**A**) and protein (**B**), merged channels in panel (**C**). Arrowheads indicate non-globular fat. Bars are 10 µm.

Consistently, the presence of fat was relevant in the aggregates of Y08 sample and was greater in larger aggregates. In particular, by separating the detection channels for fat (Figure 3A) and protein (Figure 3B), most of the fat appeared to be strongly associated with protein aggregates, indicating a direct interaction between fat globules and protein. Based on the size and appearance of fat particles (Figure 3A, arrows), fat in this product looked to be mostly unstructured, with a small proportion of intact globules, likely as an effect of a multi-processing manufacturing.

## 3. Materials and Methods

### 3.1. Chemicals

All reagents and chemicals were of analytical grade, unless differently indicated. Single standard L-amino acids were purchased from Sigma-Aldrich (St Louis, MO, USA) whereas L-Norleucine was from Alfa Aesar (Thermo Fisher Scientific, Kandel, Germany). Fast Green FCF, Nile Red and Dimethyl Sulfoxide (DMSO) were purchased from Sigma-Aldrich. Ultrapure MilliQ water (Millipore, Milford, MA, USA) was used for preparing all reagents and solutions.

### 3.2. Composition of Samples

The protein content was determined according to the Kjeldahl method [41] using the nitrogen conversion factor 6.38. Lipid content was determined by gravimetric method after solvent extraction [42], lactose content was determined by HPLC method [43], ash was determined as the weight of the incinerated sample [44], and moisture was determined using the drying oven method [45]. Samples were analyzed in triplicates.

### 3.3. Capillary Zone Electrophoresis (CZE)

All samples were dispersed (600 mg/10 mL) in 10M Urea/DTT buffer (pH 8.6) and kept at room temperature for 4 h. Solubilized samples were filtered through a 0.2 µm membrane filter (Millipore, Milan, Italy) before analysis. The CZE conditions previously described [46] were followed using a Beckman P/ACE System MDQplus, equipped with a 50 cm fused silica column (DB-WAX 126-7012, Agilent Technologies, Milan, Italy). The anode pressure injection was 0.5 psi for 20 s. Samples were run at 45 °C using a 4-min linear gradient from 0 to 30 KV, then the current voltage was kept constant at 30 KV for 56 min. Detection was carried out at 214 nm and the correct peak areas were calculated as peak area/migration time. Samples were analyzed in duplicates.

### 3.4. Amino Acid Composition

The amino acid composition was analysed on the previously hydrolyzed samples. Briefly, 50 mg powdered sample were precisely weighted in a 15-mL glass vial with a screw cap bearing a Teflon sealing valve (Mininert, VWR, Italy) and 8 mL 6N HCl was added. After bubbling with nitrogen for 2 min, the vial was sealed and kept in a thermostatic oven at 110 °C for 23 h. The hydrolysate was dried under vacuum, re-dissolved with 20 mL of 0.2 N lithium citrate buffer at pH 2.2 and quantitatively transferred into a 25-mL volumetric flask. After addition of 1 mL of the internal standard solution (Norleucine, 1.20 mg/mL), the volume was adjusted to the mark with 0.2 N tri-sodium citrate buffer at pH 2.2 to reach a final concentration of around 2 mg powder/mL. The solution was filtered on 0.2 µm membrane filter (Millipore, Milford MA) prior to injection. Free amino acids content was determined on 3 g of powder, dissolved in 40 mL of 0.2 N sodium citrate buffer at pH 2.2, under magnetic stirring for 60 min. A 10-mL aliquot of sample was then transferred into a 25-mL volumetric flask and deproteinated by dropwise adding 10 mL of 7.5% (*w*/*v*) sulfosalicylic acid at pH 1.75. After 5 min stirring, 1 mL of the internal standard solution were added, volume was adjusted to the mark with 0.2 N tri-sodium citrate buffer at pH 2.2, and the sample was filtered on 0.2 µm membrane filter. The analysis for both total and free amino acids was carried out by ion exchange chromatography (IEC) with Ninhydrin post-column derivatisation, according to the method described by Hogenboom et al. [47]. An amino acid analyser Biochrom 30+ (Erreci, Milan, Italy) was used and amino acid quantification was carried out with four-point calibration curves. Injection volume was 100 µL. Samples were analyzed in duplicates.

### 3.5. Confocal Laser Scanning Microscopy (CLSM)

In an Eppendorf vial, 450 µL of rehydrated sample (10 g/100 mL water, stirring for 2 h) were added with 50 µL of Fast Green FCF (1 mg/mL water) to stain protein, and 50 µL of Nile Red (1 mg/mL DMSO) to stain fat and kept in the dark for 5 min. A sample volume of 8 µL of the obtained preparation were transferred onto the microscope slide, covered with a coverslip and observed after sealing with nail polish [48]. The same staining procedure was performed on the rehydrated sample after addition of the urea/DTT buffer (100 µL sample + 500 µL buffer) in order to adopt reducing conditions. CLSM observations were carried out using an inverted confocal microscope (Nikon A1+, Minato, Japan). The excitation/emission wavelengths were set at 488 nm/520–590 nm for Nile Red and at 638 nm/660–740 nm for Fast Green FCF, respectively.

### 3.6. Statistical Analysis

One-way analysis of variance (ANOVA) and Tukey’s test were used to study the difference between means by using SPSS Win 12.0 program Version 27 (SPSS Inc. IBM Corp., Chicago, IL, USA). Differences at *p* < 0.05 were considered significant.

## 4. Conclusions

Whey-protein based PSS are basically mixtures of dried ingredients, namely WPC and WPI, with protein components that have already suffered some heat-damage depending on manufacturing conditions and the presence of lactose or fat. Our data evidenced that the formation of non-reducible crosslinks, and thus of insoluble aggregates may vary significantly among products. Both CZE and CLSM appeared to be suitable tools to investigate the modifications induced by technological treatments on the structure of protein and fat in PSS. CZE allowed us to detect the presence of proteins other than whey proteins or undeclared hydrolysis products. Conversely, the evaluation of TAA and FAA profiles allowed us to verify whether the claimed supplementation was complied with. The implementation of non-thermal milk sanitizing processes in the manufacturing process of PSS would also reduce irreversible modifications of the protein fraction that represents the most valuable component of these products [49]. Finally, findings from this study suggest that mandatory batch testing of commercial PSS could be advisable to ensure compliance with labelled data and explore for potential adulteration.

## Figures and Tables

**Table 1:** Composition (g/100 g) of commercial samples of powdered sport supplements (PSS) and reference samples (REF).

Samples	PSS *					REF		
	A47	C64	Y08	S15	L03	WPI	WPC	WP
Protein	84.1 ± 0.2 ^a^	76.7 ± 0.1 ^b^	66.5 ± 0.1 ^c^	95.8 ± 0.1 ^d^	86.1 ± 0.1 ^a^	88.3 ± 9.8 ^a^	35.1 ± 4.2 ^e^	13.5 ± 2.4 ^f^
Lipids	1.7 ± 0.2 ^a^	1.2 ± 0.1 ^b^	3.3 ± 0.4 ^c^	0.0 ^d^	0.0 ^d^	0.0 ^d^	3.1 ± 0.4 ^c^	1.2 ± 0.1 ^b^
Lactose	0.5 ± 0.1 ^a^	0.0 **^a^	6.7 ± 0.2 ^b^	0.0 ^a^	0.0 ^a^	0.0 ^a^	51.2 ± 1.3 ^c^	73.3 ± 1.2 ^d^
Ash	5.7 ± 0.0 ^a^	5.3 ± 0.2 ^a^	5.0 ± 0.1 ^a^	1.2 ± 0.0 ^b^	5.8 ± 0.0 ^a^	5.1 ± 0.9 ^a^	7.3 ± 1.1 ^c^	8.0 ± 1.2 ^c^
Moisture	7.1 ± 0.1 ^a^	6.8 ± 0.0 ^a^	7.3 ± 0.1 ^a^	3.1 ± 0.0 ^c^	6.9 ± 0.1 ^a^	3.4 ± 0.6 ^bc^	4.1 ± 0.6 ^b^	3.2 ± 0.5 ^c^

* A47: whey proteins obtained by ultrafiltration; C64: hydrolyzed whey protein isolate; Y08: whey protein concentrate; S15: whey protein isolate added with alanine and glutamine; L03: isolated and ultrafiltered whey proteins. ** Commercial label indicates that the powder contains 5.7 g/100 g maltodextrin. Data are presented as mean value ± standard deviation. Different superscript letters (a–f) within row indicate significant differences (*p* < 0.05; Tukey test).

**Table 2:** Content of total (TAA) and free (FAA) amino acids (g/100 g product) of commercial powdered sport supplements (PSS) and reference samples (REF). Data are the mean ± StdDev of duplicate analyses of *n* = 3 batches for each commercial PSS and of *n* = 4 batches of reference samples.

Sample	PSS										Reference					
	A47		C64		Y08		S15		L03		WPI		WPC35		WP	
	TAA	FAA	TAA	FAA	TAA	FAA	TAA	FAA	TAA	FAA	TAA	FAA	TAA	FAA	TAA	FAA
Asp	9.9 ± 0.4	----	8.9 ± 0.3	----	3.7 ± 0.1	----	7.8 ± 0.2	----	7.7 ± 0.2	----	9.8 ± 1.1	----	2.4 ± 0.4	----	0.8 ± 0.0	----
Thr	6.6 ± 0.4	----	5.8 ± 0.3	----	2.3 ± 0.1	----	5.3 ± 0.5	----	5.0 ± 0.2	----	6.7 ± 0.4	----	2.2 ± 0.3	----	0.7 ± 0.0	----
Ser	4.7 ± 0.1	----	4.1 ± 0.1	----	1.9 ± 0.0	----	3.6 ± 0.2	----	4.3 ± 0.1	----	4.3 ± 0.3	----	1.9 ± 0.0	----	0.6 ± 0.0	----
Glu	15.6 ± 0.7	----	13.8 ± 0.8	----	6.4 ± 0.3	----	21.6 ± 1.9	0.1 ± 0.0	15.2 ± 0.5	----	20.4 ± 1.5	----	5.4 ± 0.5	----	1.7 ± 0.2	----
Gln	0 ± 0.0	----	0 ± 0.0	----	0 ± 0.0	----	0 ± 0	10.4 ± 0.4	0.0 ± 0.0	----	0.0 ± 0.0	5.1 ± 0.5	0.0 ± 0.0	----	0.0 ± 0.0	----
Gly	1.6 ± 0.1	----	1.4 ± 0.1	----	7.8 ± 0.3	2.6 ± 0.1	9.3 ± 1	4.1 ± 0.2	1.4 ± 0.0	----	1.4 ± 0.2	----	0.7 ± 0.0	----	0.2 ± 0.0	----
Ala	4.7 ± 0.1	----	4.3 ± 0.1	----	2.1 ± 0.1	----	7.1 ± 0.6	3.2 ± 0.1	3.5 ± 0.1	----	4.4 ± 0.4	----	1.8 ± 0.2	----	0.6 ± 0.0	----
Val	5.8 ± 0.3	----	5.1 ± 0.3	----	2.0 ± 0.0	----	4.2 ± 0.6	----	4.9 ± 0.2	----	5.2 ± 0.4	----	2.1 ± 0.3	----	0.7 ± 0.0	----
Cys2	2.1 ± 0.3	----	1.9 ± 0.1	----	0.7 ± 0.0	----	1.5 ± 0.2	----	1.3 ± 0.0	----	2.1 ± 0.3	----	0.6 ± 0.0	----	0.1 ± 0.0	----
Met	1.9 ± 0.3	----	1.6 ± 0.0	----	0.6 ± 0.0	----	1 ± 0.8	----	1.7 ± 0.1	----	1.8 ± 0.2	----	0.7 ± 0.0	----	0.2 ± 0.0	----
Ile	6.4 ± 0.1	----	5.5 ± 0.0	----	2.1 ± 0.1	----	4.3 ± 0.2	----	4.8 ± 0.2	----	6.0 ± 0.6	----	2.2 ± 0.1	----	0.7 ± 0.0	----
Leu	9.7 ± 0.2	----	8.9 ± 0.2	0.2 ± 0.0	3.6 ± 0.2	----	7.7 ± 0.4	----	8.3 ± 0.3	----	9.2 ± 0.9	----	3.6 ± 0.4	----	1.1 ± 0.0	----
Tyr	2.5 ± 0.1	----	2.2 ± 0.1	0.2 ± 0.0	0.1 ± 0.0	----	1.9 ± 0.1	----	1.0 ± 0.0	----	2.4 ± 0.2	----	0.8 ± 0.0	----	0.2 ± 0.0	----
Phe	2.7 ± 0.1	----	2.5 ± 0.1	----	1.3 ± 0.0	----	2.1 ± 0.2	----	3.0 ± 0.1	----	2.5 ± 0.1	----	1.1 ± 0.1	----	0.3 ± 0.0	----
Lys	9.2 ± 0.5	----	8.3 ± 0.3	----	3.1 ± 0.1	----	10.4 ± 0.4	3.4 ± 0.1	7.0 ± 0.2	----	8.2 ± 0.9	----	3.2 ± 0.2	----	1.0 ± 0.1	----
His	1.5 ± 0.0	----	1.3 ± 0.0	----	0.7 ± 0.0	----	1.1 ± 0.1	----	1.6 ± 0.0	----	1.3 ± 0.2	----	0.8 ± 0.0	----	0.2 ± 0.0	----
Arg	1.9 ± 0.0	----	1.7 ± 0.0	----	1.3 ± 0.1	----	1.4 ± 0.0	----	2.4 ± 0.1	----	2.9 ± 0.1	1 ± 0.0	1.0 ± 0.0	----	0.3 ± 0.0	----
Pro	6.0 ± 0.1	----	5.0 ± 0.2	----	2.7 ± 0.1	----	4.2 ± 0.2	----	6.5 ± 0.2	----	5.4 ± 0.5		2.1 ± 0.3	----	0.7 ± 0.0	----
Total	92.6 ± 0.8	0.0 ± 0.0	82.5 ± 1	0.4 ± 0.0	43.0 ± 2.2	2.6 ± 0.1	94.6 ± 1	21.2 ± 0.8	79.6 ± 3.2	0.0 ± 0.0	94.0 ± 6.2	6.1 ± 0.8	32.5 ± 3.4	0.0 ± 0.0	10.0 ± 1.3	0.0 ± 0.0

## Data Availability

Not applicable.

## Supplementary Materials

The following supporting information can be downloaded at: https://www.mdpi.com/article/10.3390/molecules27113487/s1, Table S1: Labelled composition of commercial powdered sport supplements (PSS); Table S2: Content of total and free amino acids of commercial powdered sport supplements (PSS)

## Sample Availability

Samples of the powdered sport supplements are not available from the authors.
