# Supplementary material for: Focus on the Protein Fraction of Sports Nutrition Supplements
Source: Molecules. 2022 May 28;27(11):3487. doi: 10.3390/molecules27113487 (PMC9182466; doi:10.3390/molecules27113487)
Supplement: Supplementary file 1 [file molecules-27-03487-s001.zip › molecules-1725740-supplementary.pdf]

Supplementary Table S1. Labelled composition of commercial powdered sport supplements (PSS).

Sample	Protein (g/100g)	Lipids (g/100g)	Lactose (g/100g)	Ash (g/100g)	Moisture (g/100g)
A47	85.5	2.4	0.7	5.2	6.1
C64 *	79.3	1.4	0.0	5.4	6.4
Y08	68.2	3.8	7.2	4.8	6.7
S15	92.8	0	0	2.3	3.3
L03	87.3	0	0	6.2	5.5

* Commercial label indicates that the powder contains 5.7g/100g maltodextrin

Supplementary Table S2. Content of total (TAA) and free (FAA) amino acids (g/100g product) of commercial powdered sport supplements (PSS) and reference samples (Reference). Data are the mean ± StdDev of duplicate analyses of n=3 batches for each commercial PSS and of n=4 batches of reference samples. Different superscript letters (a-f) within row indicate significant differences ($p<0.05$; Tukey's test).

Sample	PSS										Reference					
	A47		C64		Y08		S15		L03		WPI		WPC35		WP	
	TAA	FAA	TAA	FAA	TAA	FAA	TAA	FAA	TAA	FAA	TAA	FAA	TAA	FAA	TAA	FAA
Asp	9.9 ± 0.4^{a}	----	8.9 ± 0.3^{ab}	----	3.7 ± 0.1^{c}	----	7.8 ± 0.2^{b}	----	7.7 ± 0.2^{b}	----	9.8 ± 1.1^{a}	----	2.4 ± 0.4^{c}	----	0.8 ± 0.0^{d}	----
Thr	6.6 ± 0.4^{ab}	----	5.8 ± 0.3^{ac}	----	2.3 ± 0.1^{d}	----	5.3 ± 0.5^{c}	----	5.0 ± 0.2^{c}	----	6.7 ± 0.4^{b}	----	2.2 ± 0.3^{d}	----	0.7 ± 0.0^{e}	----
Ser	4.7 ± 0.1^{a}	----	4.1 ± 0.1^{b}	----	1.9 ± 0.0^{c}	----	3.6 ± 0.2^{d}	----	4.3 ± 0.1^{ab}	----	4.3 ± 0.3^{ab}	----	1.9 ± 0.0^{c}	----	0.6 ± 0.0^{e}	----
Glu	15.6 ± 0.7^{a}	----	13.8 ± 0.8^{a}	----	6.4 ± 0.3^{b}	----	21.6 ± 1.9^{c}	0.1 ± 0.0	15.2 ± 0.5^{a}	----	20.4 ± 1.5^{c}	----	5.4 ± 0.5^{b}	----	1.7 ± 0.2^{d}	----
Gln	0.0 ± 0.0^{a}	----	0.0 ± 0.0^{a}	----	0.0 ± 0.0^{a}	----	0.0 ± 0.0^{a}	10.4 ± 0.4	0.0 ± 0.0^{a}	----	0.0 ± 0.0^{a}	5.1 ± 0.5	0.0 ± 0.0^{a}	----	0.0 ± 0.0^{a}	----
Gly	1.6 ± 0.1^{a}	----	1.4 ± 0.1^{a}	----	7.8 ± 0.3^{b}	2.6 ± 0.1	9.3 ± 1^{c}	4.1 ± 0.2	1.4 ± 0.0^{a}	----	1.4 ± 0.2^{a}	----	0.7 ± 0.0^{ad}	----	0.2 ± 0.0^{d}	----
Ala	4.7 ± 0.1^{a}	----	4.3 ± 0.1^{a}	----	2.1 ± 0.1^{b}	----	7.1 ± 0.6^{c}	3.2 ± 0.1	3.5 ± 0.1^{d}	----	4.4 ± 0.4^{a}	----	1.8 ± 0.2^{b}	----	0.6 ± 0.0^{e}	----
Val	5.8 ± 0.3^{a}	----	5.1 ± 0.3^{a}	----	2.0 ± 0.0^{b}	----	4.2 ± 0.6^{c}	----	4.9 ± 0.2^{ac}	----	5.2 ± 0.4^{a}	----	2.1 ± 0.3^{b}	----	0.7 ± 0.0^{d}	----
Cys2	2.1 ± 0.3^{a}	----	1.9 ± 0.1^{ab}	----	0.7 ± 0.0^{c}	----	1.5 ± 0.2^{bd}	----	1.3 ± 0.0^{d}	----	2.1 ± 0.3^{a}	----	0.6 ± 0.0^{c}	----	0.1 ± 0.0^{e}	----
Met	1.9 ± 0.3^{a}	----	1.6 ± 0.0^{a}	----	0.6 ± 0.0^{b}	----	1 ± 0.8^{ab}	----	1.7 ± 0.1^{a}	----	1.8 ± 0.2^{a}	----	0.7 ± 0.0^{b}	----	0.2 ± 0.0^{b}	----
Ile	6.4 ± 0.1^{a}	----	5.5 ± 0.0^{b}	----	2.1 ± 0.1^{c}	----	4.3 ± 0.2^{d}	----	4.8 ± 0.2^{d}	----	6.0 ± 0.6^{ab}	----	2.2 ± 0.1^{c}	----	0.7 ± 0.0^{e}	----
Leu	9.7 ± 0.2^{a}	----	8.9 ± 0.2^{ab}	0.2 ± 0.0	3.6 ± 0.2^{c}	----	7.7 ± 0.4^{d}	----	8.3 ± 0.3^{bd}	----	9.2 ± 0.9^{ab}	----	3.6 ± 0.4^{c}	----	1.1 ± 0.0^{e}	----
Tyr	2.5 ± 0.1^{a}	----	2.2 ± 0.1^{b}	0.2 ± 0.0	0.1 ± 0.0^{c}	----	1.9 ± 0.1^{d}	----	1.0 ± 0.0^{e}	----	2.4 ± 0.2^{ab}	----	0.8 ± 0.0^{e}	----	0.2 ± 0.0^{c}	----
Phe	2.7 ± 0.1^{a}	----	2.5 ± 0.1^{a}	----	1.3 ± 0.0^{b}	----	2.1 ± 0.2^{c}	----	3.0 ± 0.1^{d}	----	2.5 ± 0.1^{a}	----	1.1 ± 0.1^{b}	----	0.3 ± 0.0^{e}	----
Lys	9.2 ± 0.5^{a}	----	8.3 ± 0.3^{a}	----	3.1 ± 0.1^{b}	----	10.4 ± 0.4^{c}	3.4 ± 0.1	7.0 ± 0.2^{d}	----	8.2 ± 0.9^{a}	----	3.2 ± 0.2^{b}	----	1.0 ± 0.1^{e}	----
His	1.5 ± 0.0^{ab}	----	1.3 ± 0^{bd}	----	0.7 ± 0.0^{c}	----	1.1 ± 0.1^{d}	----	1.6 ± 0.0^{a}	----	1.3 ± 0.2^{bd}	----	0.8 ± 0.0^{c}	----	0.2 ± 0.0^{e}	----
Arg	1.9 ± 0.0^{a}	----	1.7 ± 0^{a}	----	1.3 ± 0.1^{b}	----	1.4 ± 0^{b}	----	2.4 ± 0.1^{c}	----	2.9 ± 0.1^{d}	1 ± 0.0	1.0 ± 0.0^{e}	----	0.3 ± 0.0^{f}	----
Pro	6.0 ± 0.1^{ab}	----	5.0 ± 0.2^{c}	----	2.7 ± 0.1^{d}	----	4.2 ± 0.2^{e}	----	6.5 ± 0.2^{a}	----	5.4 ± 0.5^{bc}		2.1 ± 0.3^{d}	----	0.7 ± 0.0^{f}	----
Total	92.6 ± 0.8^{a}	0.0 ± 0.0	82.5 ± 1^{b}	0.4 ± 0.0	43.0 ± 2.2^{c}	2.6 ± 0.1	94.6 ± 1^{a}	21.2 ± 0.8	79.6 ± 3.2^{b}	0.0 ± 0.0	94.0 ± 6.2^{a}	6.1 ± 0.8	32.5 ± 3.4^{d}	0.0 ± 0.0	10.0 ± 1.3^{e}	0.0 ± 0.0
